# Plant cell wall remodeling and peptide signaling under abiotic and biotic stress

**DOI:** 10.1016/j.xplc.2026.101741

**Published:** 2026-01-29

**Authors:** Joy Debnath, Richard Noi Morton, Timo Engelsdorf, Nora Gigli-Bisceglia

**Affiliations:** 1 Plant Stress Resilience, Institute of Environmental Biology, Utrecht University, Utrecht, the Netherlands; 2 Molecular Plant Physiology, Department of Biology, Philipps-Universität Marburg, 35043 Marburg, Germany

**Keywords:** plant cell wall, cell wall integrity, peptide signaling, temperature stress, osmotic stress, salinity, plant–pathogen interaction

## Abstract

Plants are continuously exposed to abiotic and biotic stresses, often in combination, necessitating tightly coordinated metabolic and structural adaptations. A central component of these responses is the plant cell wall, a dynamic extracellular matrix that undergoes extensive remodeling to maintain integrity under stress conditions. Both abiotic and biotic cues trigger modifications in cell wall composition and architecture, which in turn shape signaling pathways and defense mechanisms. Although hormonal signaling has long been recognized as a key driver of stress adaptation, accumulating evidence points to a crucial role for small signaling peptides (SSPs) in modulating stress responses. Typically, fewer than 100 amino acids in length, SSPs function through diverse mechanisms, including transcriptional regulation and direct interactions with cell wall components. In this review, we examine the interplay between environmental stress, cell wall remodeling, and SSP-mediated signaling. We provide an overview of stress-specific cell wall modifications and outline how SSPs participate in these responses. Through exploratory analyses of published transcriptomic datasets, we illustrate how SSP precursor expression patterns may indicate potential roles in cell wall-mediated stress adaptation. Together, we conclude that SSP signaling constitutes an integral component of plant responses to abiotic and biotic stress and highlight key directions for future functional studies aimed at elucidating the roles of SSPs in cell wall remodeling.

## Teaser

Plant cell walls are highly dynamic structures that undergo extensive remodeling in response to abiotic and biotic stresses. This review examines how stress-induced cell wall modifications are integrated with signaling pathways mediated by small signaling peptides. It highlights emerging roles of peptide signaling in coordinating cell wall integrity, stress responses, and adaptive growth.

## Introduction

Abiotic stresses often trigger modifications in cell wall composition, affecting mechanical properties and altering cell wall-dependent signaling (Le Gall et al., 2015; Tenhaken, 2015; Rui and Dinneny, 2020). These changes can strengthen the wall to protect against mechanical damage or modify its elasticity to accommodate changes in water availability (Clifford et al., 1998; Le Gall et al., 2015; Tenhaken, 2015). Similarly, biotic interactions induce significant cell wall modifications (Munzert and Engelsdorf, 2025; Pinto et al., 2025). Many filamentous pathogens rely on breaching the plant cell wall to modify host metabolism and signaling pathways and/or to access intracellular nutrients (Faulkner and Robatzek, 2012; Meisrimler et al., 2021). While in some cases pathogen-derived enzymes actively degrade cell wall components, in others the plant itself modifies its cell wall architecture in response to infection, either reinforcing the barrier against invasion or allowing for controlled cell wall loosening (Munzert and Engelsdorf, 2025).

The adaptive processes outlined above are controlled by a wide network of signaling pathways. These pathways prominently include phytohormone signaling (Aerts et al., 2021; Waadt et al., 2022); however, emerging evidence suggests that, in addition, small signaling peptides (SSPs) play a crucial role in coordinating cell wall-related stress responses (Narváez-Vásquez et al., 2005; Wolf et al., 2014; Engelsdorf et al., 2018; Moussu et al., 2023; Liu et al., 2024a; Rößling et al., 2024; Schoenaers et al., 2024; Zhai et al., 2024). Despite this progress, it remains difficult to determine which SSPs merely occur in the apoplast to mediate signaling between neighboring cell membranes and which SSPs interact with cell wall polymers or are actively involved in responses to altered cell wall properties. Cell wall modifications and peptide signaling events have largely been studied in isolation, and the underlying molecular intersections are only beginning to emerge. This separation has led to the perception of two largely independent research fields—“cell wall biology” and “peptide signaling”—rather than a unified framework. The goal of this review is to synthesize current knowledge at the intersection of cell wall remodeling and peptide signaling in plants exposed to abiotic and biotic stress. We highlight (1) stress-specific wall modifications, (2) peptides that contribute to these adaptive responses, and (3) known or hypothesized molecular mechanisms connecting the two systems. In addition, we illustrate how publicly available transcriptomic resources can be reanalyzed to generate testable hypotheses regarding peptide regulation under stress. By combining established evidence with exploratory insights, we aim to outline not only what is currently known but also where the major knowledge gaps lie and how future research may bridge them.

## The plant cell wall: A dynamic network of carbohydrates and structural proteins

The plant cell wall provides structural integrity yet remains remarkably plastic. Both these properties are essential for the roles of cell walls in mediating intercellular communication and regulating cellular expansion. The main components of the cell wall are complex polysaccharides, which are organized into three major types: pectin, cellulose, and hemicellulose (Delmer et al., 2024). Together with structural proteins and aromatic polymers such as lignin and suberin, these polysaccharides constitute an intricate and dynamic network capable of maintaining a wide range of mechanical properties under diverse developmental and environmental conditions (Gibson, 2012). In dicots, the primary cell wall contains a relatively low cellulose content (below 20%), yet this amount is sufficient to confer mechanical strength and support (Gigli-Bisceglia et al., 2020). The stiffness of cellulose arises from its highly ordered crystalline structure. Cellulose consists of parallel β-1,4-D-glucan chains, synthesized by the CELLULOSE SYNTHASE COMPLEX (CSC) at the plasma membrane. CELLULOSE SYNTHASE (CESA) proteins initially assemble into trimers, which subsequently form a hexameric CSC containing 18 CESA subunits (Polko and Kieber, 2019). However, recent structural and biophysical studies suggest that CSC composition may be more heterogeneous, and that secondary cell wall CSCs in particular may include 24 subunits, with other multimeric states (e.g., 18, 30, or 36 subunits) not entirely ruled out (Tai et al., 2023). Cellulose synthesis in *Arabidopsis thaliana* (hereafter *Arabidopsis*) primary cell walls is mediated predominantly by CESA1, CESA3, and CESA6, whereas secondary cell walls rely on the function of CESA4, CESA7, and CESA8 (Polko and Kieber, 2019). Within secondary walls, cellulose can constitute up to 80% of cell wall polymers, and cellulose microfibrils are extensively crosslinked by hemicelluloses, forming an integrated load-bearing matrix (Kumar et al., 2016; Delmer et al., 2024).

Pectins are the most abundant cell wall components of primary dicot cell walls, with homogalacturonan (HG)—a linear polymer composed of α-1,4-D-galacturonic acid residues—constituting the major structural domain (approximately 65% of total pectin; Atmodjo et al., 2013). Most of the remaining pectin consists of two types of branched pectins: rhamnogalacturonan-I (RG-I) and rhamnogalacturonan-II (RG-II). RG-I comprises a backbone of alternating galacturonic acid and rhamnose residues with side chains of arabinan, galactan, and arabinogalactan (Kaczmarska et al., 2022). In contrast, RG-II contains an HG backbone with four to six structurally diverse side chains (Hays et al., 2025). Evidence from enzymatic cell wall digestion indicates that HG forms heteroglycan structures with both RG-I and RG-II. RG-II can form dimers through borate diester complex formation, potentially leading to crosslinking of pectin heteroglycans and thereby regulating cell wall mechanical properties (Delmer et al., 2024). Recent work shows that reduced RG-II dimerization disrupts cell wall mechanics and, in turn, alters auxin transport and brassinosteroid signaling, leading to defects in differential growth (Jewaria et al., 2025). This finding highlights RG-II crosslinking as a key integrator of wall structure and hormone-mediated growth regulation. HG is initially synthesized in a methylesterified form within the Golgi apparatus before undergoing controlled demethylesterification in the apoplast (Anderson and Pelloux, 2025). This post-synthetic modification is mediated by two enzyme families with opposing functions: PECTIN METHYLESTERASEs (PMEs; 67 in *Arabidopsis*) and proteinaceous PME INHIBITORs (PMEIs, 69 in *Arabidopsis*), which jointly modulate HG function during development and environmental stress (Levesque-Tremblay et al., 2015). Nearly 70% of PME proteins in *Arabidopsis* contain N-terminal PMEI-like PRO domains and subtilase cleavage motifs in the linker region between the PRO and PME domains, which can regulate PME activity under stress conditions (Del Corpo et al., 2020; Coculo et al., 2023). The degree of HG methylesterification has significant implications for cell wall mechanics (Peaucelle et al., 2011; Höfte et al., 2012). For a long time, lower levels of methylesterification resulting from PME activity have been associated with enhanced wall rigidity, owing to an increased number of negatively charged carboxyl groups that can be crosslinked by Ca^2+^. The resulting “egg-box” structures stabilize the cell wall, restrict expansion, and influence cellular morphology (Morris et al., 1982; Zdunek et al., 2021). Recent studies using super-resolution microscopy suggest that the relationship between HG methylation and wall extensibility is not strictly linear (Haas et al., 2020; Cosgrove, 2022). New evidence indicates that Ca^2+^ crosslinking may follow a “zipper” rather than a classical egg-box arrangement, with important consequences for wall porosity and mechanics (Obomighie et al., 2025). HG methylation plays a role in shaping the overall structure of pectin, which in turn influences cell swelling. Haas et al. (2020) proposed the “expanding beam model” to explain this process. According to this model, when specific regions of HG lose their methyl groups, HG nanofilaments undergo structural changes that cause them to shift between tightly packed and more loosely organized states, likely leading to radial swelling. Notably, although HG methylation affects filament width, the degree of expansion appears to be dictated more by structural rearrangements of pectin domains than by absolute methylation levels alone (Haas et al., 2021). PMEs and PMEIs are central regulators of cell elongation, yet deciphering their precise contributions remains challenging. The contrasting observations that PME-mediated pectin de-esterification can either promote wall expansion or enhance wall rigidity through increased calcium crosslinking indicate that pectin methylation serves multiple functions (Gallemí et al., 2022). The former effect may be facilitated by POLYGALACTURONASE (PG)-dependent HG hydrolysis (Wakabayashi et al., 2003), whereas the latter appears to depend on the concentration of free calcium ions in the apoplast, with gel stiffness decreasing when calcium crosslinks dissociate (Tibbits et al., 1998). The interplay among PME activity, calcium dynamics, and pectin structural remodeling therefore suggests a complex, context-dependent regulatory system. This remains an area of active research (Obomighie et al., 2025), with ongoing debate surrounding the precise molecular mechanisms governing cell wall extensibility versus rigidity.

In *Arabidopsis* primary cell walls, hemicellulose is mainly composed of xyloglucan (XyG), a polymer comprising a β-1,4-glucan backbone decorated with xylose, galactose, and fucose residues (Scheller and Ulvskov, 2010). Genetic studies in *Arabidopsis* have demonstrated that the CELLULOSE SYNTHASE-LIKE C (CSLC) family is responsible for synthesizing the XyG glucan backbone (Julian and Zabotina, 2022). For a long time, XyG was hypothesized to tether cellulose microfibrils, with XYLOGLUCAN ENDOTRANSGLUCOSYLASE/HYDROLASE (XTH) enzymes regulating cell wall properties by controlling XyG-mediated crosslinking between cellulose fibrils (Rose et al., 2002; Van Sandt et al., 2007). This “tethered network” model has been challenged by more recent studies (Bashline et al., 2014; Cosgrove, 2022). An emerging consensus suggests that XyG preferentially binds to the hydrophobic surfaces of cellulose microfibrils, forming localized “biomechanical hotspots” where expansins regulate the loosening of polymer interactions (Park and Cosgrove, 2012; Cosgrove, 2018; Zheng et al., 2018). These hotspots, together with the pectin–cellulose continuum, are thought to influence cell wall integrity (CWI) and extensibility (Cosgrove, 2022). The importance of XyG for cell wall-dependent responses and overall cell wall functionality was questioned by the observation that the quintuple *cslc* mutant, which lacks detectable XyG, displays only minor developmental defects (Kim et al., 2020). However, more recent work by Bou Daher et al. (2024) showed that XyG-deficient seedlings exhibit reduced shoot emergence and impaired root penetration through thick layers of sand or hard agar. Notably, these mutants are also characterized by reduced cellulose crystallinity and increased pectin levels, suggesting activation of a sensing mechanism that triggers compensatory changes in cell wall composition. Although the absence of XyG leads to weakened cell walls, normal growth is largely maintained. This effect has been attributed to reduced turgor pressure, which prevents wall rupture, and to the preservation of elastic asymmetry factors that together support directional growth despite altered wall mechanics. These findings revise our understanding of XyG function, revealing that while XyG is not strictly essential for growth—likely due to compensatory mechanisms within the cell wall—it plays a crucial role in modulating wall mechanics and remains essential for plant adaptation and stress resistance.

Glycoproteins play an important role in the structural maintenance of the plant cell wall, which is essential for its integrity, stability, and function (Nguema-Ona et al., 2014). They are involved in crosslinking cell wall components, regulating cell wall expansion, and mediating cell–cell adhesion (Marzol et al., 2018; Mishler-Elmore et al., 2021). HYDROXYPROLINE-RICH GLYCOPROTEINs (HRGPs) are predominantly found in the primary cell wall (Johnson et al., 2017). These highly glycosylated proteins form extensive networks that provide structural support and resist excessive stretching during cell growth. Extensins comprise characteristic Ser-Pro_3-5_ motifs, which are present in LEUCINE-RICH REPEAT EXTENSINs (LRXs) and PROLINE-RICH EXTENSIN-LIKE RECEPTOR KINASEs (PERKs), thereby forming chimeric extensins with signaling functions (Herger et al., 2019). Another major class of HRGPs is represented by ARABINOGALACTAN PROTEINs (AGPs). AGPs are highly glycosylated with arabinogalactan polysaccharides, which may also include glucuronic acid and rhamnose residues (Ma and Johnson, 2023). AGPs interact with RG-I glycans, forming complexes and molecular crosslinks that reinforce the cell wall network (Tan et al., 2013, 2023; Ma and Johnson, 2023). For example, SALT OVERLY SENSITIVE 5 (SOS5), a FASCICLIN-LIKE-AGP (FLA4), interacts with pectin to mediate mucilage adherence in *Arabidopsis* seeds, thereby ensuring cohesion in hydrated environments (Griffiths et al., 2014). Although the precise roles of AGPs remain incompletely understood, recent findings show that glucuronic acid residues on AGPs can bind calcium ions *in vitro* (Lopez-Hernandez et al., 2020). It has been hypothesized that reduced apoplastic pH may cause protonation of glucuronic acid residues, leading to calcium release into the apoplast (Lamport and Várnai, 2013; Lopez-Hernandez et al., 2020). Mutants in β-GLUCURONYLTRANSFERASEs, which lack glucuronidation of arabinogalactans, exhibit reduced calcium-binding capacity and display overall stunted growth phenotypes that can be alleviated by exogenous calcium application (Lopez-Hernandez et al., 2020). Similarly, while knockout mutants of single AGPs show no obvious phenotypes, higher-order mutants reveal that AGPs are essential for proper cell expansion (Pereira et al., 2016; Leszczuk et al., 2019).

While the primary role of the cell wall is to sustain cell growth, shape, and internal pressure, the balance between rigidity and flexibility can be disrupted by stress exposure and must therefore be precisely monitored and regulated. Even under physiological conditions that promote cell wall loosening, such as cell elongation, interactions among carbohydrate polymers must remain tightly controlled to ensure proper plant growth (Barnes and Anderson, 2018). Plants have evolved mechanisms that likely sense and regulate CWI (Vaahtera et al., 2019). Maintenance of CWI appears to require the coordinated action of RECEPTOR-LIKE KINASEs (RLKs), RECEPTOR-LIKE PROTEINs (RLPs), plasma membrane–localized channels, and intracellular regulators (Baez et al., 2022; Wolf, 2022). Among the 17 members of the *Catharanthus roseus* RLK1-LIKE (*Cr*RLK1L) family in *Arabidopsis*, several have been shown to play roles in CWI signaling (Vaahtera et al., 2019; Zhu et al., 2021). These proteins contain extracellular malectin-like domains that act as *bona fide* receptors for the RAPID ALKALINIZATION FACTOR (RALF; 37 members in *Arabidopsis*) peptide family (Zhu et al., 2021). In parallel, several *Cr*RLK1Ls also bind cell wall components. For instance, FERONIA (FER) preferentially binds de-methylesterified pectin to activate the RHO GTPase ROP6, thereby controlling cortical microtubule organization (Lin et al., 2022). Similarly, *in vitro* assays suggest that BUDDHA’S PAPER SEAL 1 (BUPS1), ANXUR 1 and 2 (ANX1 ANX2) can also bind pectin (Feng et al., 2018). ANX1/2 and BUPS1/2 are essential for preventing premature pollen tube rupture during fertilization (Ge et al., 2017; Baillie et al., 2024). This RALF-mediated mechanism fine-tunes cell wall degradation and maintenance, ensuring the timely and controlled release of sperm cells during pollination (Ge et al., 2017, 2019). LRX proteins can also associate with RALF peptides to modulate growth and stress responses (Mecchia et al., 2017; Moussu et al., 2020). However, whether LRXs and RALFs form larger multiprotein complexes with *Cr*RLK1Ls remains unclear (Zhang et al., 2020a). FER forms heterocomplexes with LORELEI (LRE)-LIKE GLYCOSYLPHOSPHATIDYLINOSITOL (GPI)-ANCHORED PROTEIN 1 (LLG1) and RALF23 to regulate RLK nanodomain organization at the plasma membrane, pectin methylation, and cell morphology (Xiao et al., 2019; Gronnier et al., 2022; Biermann et al., 2025). The *Cr*RLK1L THESEUS 1 (THE1) was initially identified as a mediator of hypocotyl elongation defects in a *cesa6* mutant and has since been recognized as a key sensor of responses to cellulose biosynthesis inhibition (CBI), although a cell wall ligand of THE1 has not yet been identified (Hématy et al., 2007; Vaahtera et al., 2019). THE1 regulates cell wall stiffness and hormone signaling but is dispensable for CBI-induced inhibition of cell cycle progression (Engelsdorf et al., 2018; Gigli-Bisceglia et al., 2018; Bacete et al., 2022). This observation suggests the existence of at least two distinct branches within the cellulose-deficiency response pathway: one controlled by THE1 and another that operates independently of it. In addition, THE1 binds RALF34 to control lateral root primordia emergence, and RALF34–THE1-mediated signaling has been proposed to form part of the regulatory network coordinating CWI and cell division during lateral root initiation (Gonneau et al., 2018).

## Small signaling peptides and their role in regulating cell wall-dependent mechanisms

**Figure 1:**
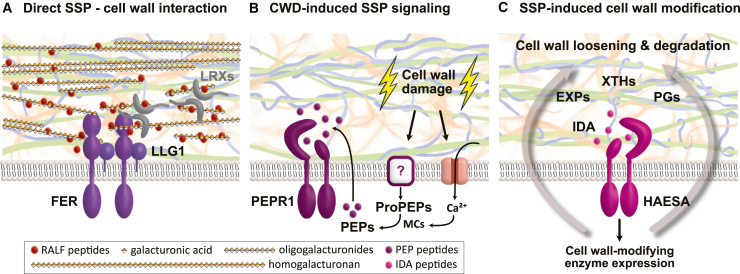
Small signaling peptides interact with cell walls in multiple ways. Three examples illustrate how small signaling peptides (SSPs) interact with cell wall polymers and function as integral components of cell wall-dependent processes in *Arabidopsis*. **(A)** RAPID ALKALINIZATION FACTOR (RALF) peptides directly bind de-methylesterified homogalacturonan and oligogalacturonides, as well as the receptor FERONIA (FER) and LRR-EXTENSINs (LRXs) (Mecchia et al., 2017; Zhao et al., 2018; Herger et al., 2020; Lin et al., 2022; Moussu et al., 2023; Rößling et al., 2024). RALF–pectin interactions can induce phase separation, recruiting FER and its co-receptor LORELEI (LRE)-LIKE GLYCOSYLPHOSPHATIDYLINOSITOL (GPI)-ANCHORED PROTEIN 1 (LLG1) into condensates upon stress exposure (Liu et al., 2024a). **(B)** Cell wall damage induces the expression of *ProPEP* genes and triggers Ca^2+^ signaling (Denness et al., 2011; Engelsdorf et al., 2018). Ca^2+^-activated METACASPASEs (MCs) catalyze the release of PLANT ELICITOR PEPTIDEs (PEPs), which interact with PEP RECEPTOR 1 (PEPR1) to regulate stress signaling (Hander et al., 2019; Shen et al., 2019). **(C)** Perception of the INFLORESCENCE DEFICIENT IN ABSCISSION (IDA) peptide by the receptor HAESA induces the expression of cell wall-modifying enzymes, including EXPANSINs (EXPs), XYLOGLUCAN ENDOTRANSGLUCOSYLASE/HYDROLASE proteins (XTHs), and POLYGALACTURONASEs (PGs), thereby promoting cell wall loosening and degradation during abscission and lateral root development (Butenko et al., 2003; Stenvik et al., 2008; Kumpf et al., 2013; Hohmann et al., 2018).

The plant cell wall is not only a structural barrier but also a dynamic interface that facilitates the exchange of signaling molecules through the apoplast continuum. These include glycans of plant and microbial origin (Molina et al., 2022) as well as SSPs, which represent a major class of secreted signals that, despite their short length, display remarkable functional diversity (Zhang et al., 2025a). In *Arabidopsis*, several peptide families have been identified that act in both development and stress responses. Some SSPs primarily influence developmental processes, including cell-fate determination, cell proliferation, root patterning, and shoot growth (Matsubayashi and Sakagami, 1996; Ohyama et al., 2009; Fernandez et al., 2013; Ghorbani et al., 2014). Others are more strongly associated with stress adaptation, particularly in connection with cell wall impairment. Figure 1 highlights three examples of SSP–cell wall interactions that illustrate a remarkable mechanistic diversity, ranging from direct SSP–cell wall attachment to signaling processes in which either the cell wall or SSPs act upstream of the other. One of the best-characterized families is that of RALF peptides, which not only act as bona fide ligands of *Cr*RLK1L protein kinases but also bind to demethylesterified HG to regulate cell wall properties (Figure 1A) (Gonneau et al., 2018; Ge et al., 2019; Abarca et al., 2021; Moussu et al., 2023; Liu et al., 2024b; Rößling et al., 2024; Schoenaers et al., 2024). Other peptide families regulate wall-dependent processes more indirectly. CLAVATA3/EMBRYO SURROUNDING REGION (CLE) peptides modulate stomatal aperture (Yamaguchi et al., 2016; Zhang et al., 2020b; Willoughby and Nimchuk, 2021). This mechanism depends on the reversible elasticity of guard-cell walls, which enables stomata to open under high turgor pressure and close under low turgor pressure (Jaafar and Anderson, 2024). Related families, such as the EPIDERMAL PATTERNING FACTORs (EPFs; 11 members in *Arabidopsis*) and EPF-LIKE peptides (EPFLs; 9 members in *Arabidopsis*), act via the ERECTA and ERECTA-LIKE 1 receptors to regulate stomatal development and distribution (Hunt et al., 2010; Sugano et al., 2010; Herrmann and Torii, 2020).

PLANT ELICITOR PEPTIDE (PEP) family SSPs amplify defense signaling upon stress exposure and act as negative regulators of cell wall-damage responses (Klauser et al., 2015; Engelsdorf et al., 2018). *Arabidopsis* encodes eight PEP precursors (ProPEPs), seven of which (ProPEPs 1, 2, 3, 4, 5, 7, and 8) undergo proteolytic maturation by Ca^2+^-activated METACASPASEs (MCs) (Hander et al., 2019; Shen et al., 2019). For ProPEP1–ProPEP4, this process is associated with the covalent addition of the small ubiquitin-related modifier (SUMO), a modification known as SUMOylation. In particular, SUMOylation of ProPEP1 is required for its association with MC4, thereby promoting its processing and the release of PEP1 for downstream stress responses (Zhang et al., 2025b). In their mature forms, PEPs are recognized by the receptors PEP RECEPTOR 1 (PEPR1) and PEPR2 (Yamaguchi et al., 2006, 2010). *ProPEP* expression is strongly induced by wall-associated stresses, including wounding, pathogen attack, salinity, and even by specific cell wall damage caused by cellulose biosynthesis inhibition (Bartels and Boller, 2015; Engelsdorf et al., 2018; Nakaminami et al., 2018). Thus, PEPs exemplify how cell wall-derived cues can trigger SSP signaling (Figure 1B). Interestingly, exogenous PEP1 treatment induces ectopic lignification via PEPR2 (Engelsdorf et al., 2018), although its precise role in cell wall metabolism remains unresolved. SERINE-RICH ENDOGENOUS PEPTIDEs (SCOOPs; approximately 50 members in *Arabidopsis*) constitute another large SSP family that is connected to cell wall damage responses, as well as regulation of immunity, flowering, and senescence by signaling through the MALE DISCOVERER 1-INTERACTING RECEPTOR-LIKE KINASE 2 (MIK2) (Hou et al., 2021; Rhodes et al., 2021; Zhang et al., 2024). While senescence requires tightly controlled cell wall degradation, direct involvement of SCOOPs in wall metabolism remains unclear. However, several *ProSCOOP* genes are differentially expressed upon cellulose biosynthesis inhibition, and SCOOP18 has been linked to specific cell wall-damage responses (Zhai et al., 2024).

In developmental contexts, hydrolysis of cell wall components is essential for organ separation during abscission (Lewis et al., 2006; Pautot et al., 2025). This process is orchestrated by INFLORESCENCE DEFICIENT IN ABSCISSION (IDA) and IDA-LIKE (IDL) peptides, which bind the HAESA (HAE) and HAESA-LIKE 2 (HSL2) receptors and recruit SOMATIC EMBRYOGENESIS RECEPTOR KINASEs (SERKs), thereby inducing pectinases and other wall-modifying enzymes in the abscission zone (Butenko et al., 2003; Stenvik et al., 2008; Kumpf et al., 2013; Hohmann et al., 2018). In addition to floral abscission, the IDA–HAE module regulates pectin degradation during lateral root emergence (Kumpf et al., 2013) and exemplifies how SSP-induced signaling can function upstream of cell wall modification (Figure 1C). PHYTOSULFOKINEs (PSKs), encoded by seven genes (5 canonical) in *Arabidopsis*, regulate cell proliferation through the PSK RECEPTOR 1 (PSKR1) and PSKR2 kinases (He et al., 2024). In carrot protoplasts, PSK treatment alters pectin and AGP composition, suggesting a direct impact on cell wall organization (Godel-Jędrychowska et al., 2019). Some SSPs also control the spatial deposition of wall barriers. CASPARIAN STRIP INTEGRITY FACTORs (CIFs; 5 members in *Arabidopsis*) are perceived by the SCHENGEN 3/GASSHO 1 (SGN3/GSO1) and GSO2 receptor kinases, ensuring correct lignin and suberin deposition in the root endodermis (Pfister et al., 2014; Nakayama et al., 2017). Loss of CIF signaling results in discontinuous Casparian strips and impaired nutrient homeostasis. Finally, the C-TERMINALLY ENCODED PEPTIDEs (CEPs; 12 members in *Arabidopsis*) regulate primary and lateral root development by signaling through CEP RECEPTOR 1 (CEPR1) (Taleski et al., 2023; Chapman et al., 2024). Although CEP-mediated inhibition of root growth is partly dependent on cytokinin biosynthesis and associated with auxin transport, a direct link to cell wall remodeling has not yet been demonstrated. Interestingly, mutants impaired in cytokinin biosynthesis are less sensitive to CEP-induced inhibition of root growth (Taleski et al., 2023), whereas cellulose impairment induces trans-zeatin (tZ) accumulation, and application of tZ suppresses the effects of cell wall damage on the expression of cell-cycle-related genes (Gigli-Bisceglia et al., 2018).

Taken together, these examples illustrate that SSPs contribute to a broad spectrum of processes that depend on cell wall remodeling. This diversity raises an important question: to what extent are SSPs involved in the regulation of CWI, and to what extent do they function as signals in specific developmental or stress contexts? While individual case studies point to well-defined peptide–wall modules, much of the available evidence remains fragmented, and many proposed connections are still hypothetical. To better understand this interplay in the context of stress-induced cell wall remodeling, it is therefore useful to examine how abiotic and biotic stresses impact the plant cell wall and how specific peptide families are recruited under these conditions. In the following sections, we synthesize current knowledge of cell wall remodeling under different stress scenarios, highlighting where peptide signaling has been implicated, where evidence is still lacking, and how these insights may ultimately converge into a unified framework of cell wall–peptide crosstalk.

## Cell wall remodeling and peptide signaling under abiotic and biotic stresses

**Figure 2:**
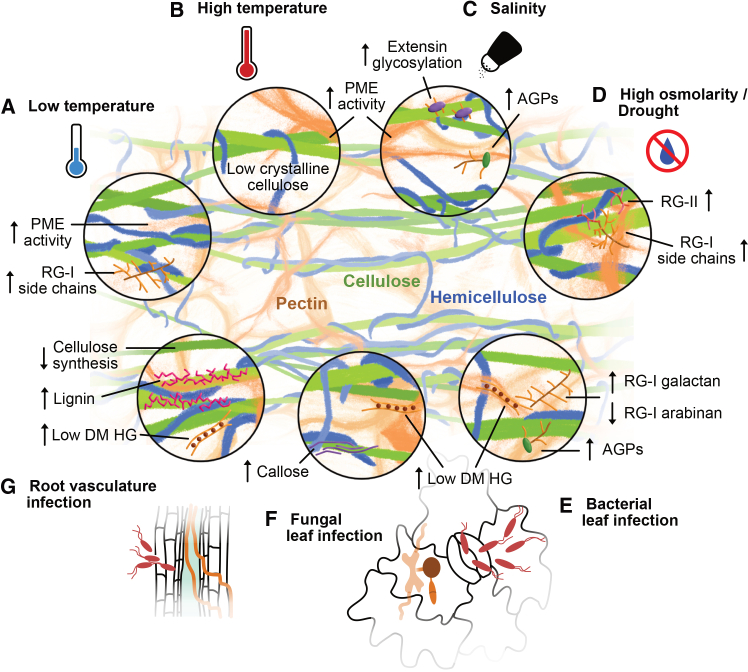
Cell wall architecture is affected by abiotic and biotic stress. The plant cell wall is primarily composed of three major polysaccharide groups—cellulose, hemicellulose, and pectin—which form a dynamic network through direct and indirect interactions. Magnified insets highlight specific regions of this network and illustrate stress-induced changes in cell wall composition and remodeling. **(A)** Low temperature increases Pectin methylesterase (PME) activity in *Arabidopsis* and promotes rhamnogalacturonan-I (RG-I) side-chain accumulation in spinach, Japanese mustard spinach, and crown daisy (Qu et al., 2011; Chen et al., 2018; Takahashi et al., 2024), potentially enhancing pectin–pectin crosslinking and limiting cell expansion (Penfield, 2008). **(B)** High temperature also upregulates PME activity and reduces cellulose crystallinity in *Arabidopsis* (Fujita et al., 2013; Huang et al., 2017; Wu et al., 2022). **(C)** Salt stress in *Arabidopsis* induces PME activity (Gigli-Bisceglia et al., 2022), increases Arabinogalactan protein (AGP) abundance (Lamport et al., 2006), and enhances extensin glycosylation (Zou et al., 2024). **(D)** Drought and osmotic stress stimulate RG-I accumulation in wheat (Leucci et al., 2008) and enhance RG-I and RG-II remodeling in drought-tolerant soybean genotypes (Coutinho et al., 2021). **(E)** Bacterial leaf infection (e.g., via stomata) increases low–degree-of-methylesterification (DM) homogalacturonan (HG) in pepper, common bean, and *Arabidopsis* (An et al., 2008; Bethke et al., 2014; De la Rubia et al., 2024). In *Arabidopsis*, this response is accompanied by increased PME activity (Bethke et al., 2014) and elevated AGP levels (Kim et al., 2023). RG-I side-chain composition is altered after infection, with increased galactan content and reduced arabinan abundance (Kim et al., 2023). **(F)** In *Arabidopsis*, fungal leaf infection induces pectin modification and accumulation of low-DM HG (Lionetti et al., 2007, 2017; Huerta et al., 2023). Penetration of the epidermal cell wall is associated with localized deposition of callose-rich papillae (Voigt, 2014). **(G)** Root vascular tissue infection by bacteria and fungi is typically associated with enhanced deposition of lignin and ligno–suberin heteropolymers in both *Arabidopsis* and tomato (Menna et al., 2021; Kashyap et al., 2022; Fan et al., 2024; Wang et al., 2024b). In *Arabidopsis,* root vascular fungal infection can also influence cellulose content through inhibition of cellulose synthesis (Menna et al., 2021) and cellulose degradation (Gámez-Arjona et al., 2022).

Plants are continuously challenged by abiotic stresses such as extreme temperatures, drought, and salinity, which alter cell wall metabolism and restrict growth. At the same time, biotic stresses imposed by pathogens threaten plant survival by directly damaging or degrading the cell wall. In the following sections, we examine how abiotic and biotic stresses drive specific modifications of the cell wall, with a focus on changes in pectin, cellulose, and cell wall-localized proteins (Figure 2 and Table 1).

**Table 1:** Stress-dependent modifications of plant cell wall components

Cell wall components	Stress	Stress-triggered modification	References
Pectin: homogalacturonan (HG) and rhamnogalacturonan (RG-I, RG-II)	Low temperature	• PME activity is induced, and HG demethylation is linked to BR signaling. *pme41* mutants are freezing sensitive. Overexpression of AtPMEI13, or expression of the alpine plant *Chorispora bungeana* PMEI1 in *Arabidopsis*, leads to freezing sensitivity	• Qu et al. (2011), Chen et al. (2018), Takahashi et al. (2019)
		• Accumulation of β-1,4-galactans and arabinose-rich RG-I side chains (*Arabidopsis*, spinach, Japanese mustard spinach, and crown daisy). *Arabidopsis gals1*/*2*/*3* triple mutants show impaired freezing tolerance	• Takahashi et al. (2024)
		• RG-II crosslinking is critical for freezing tolerance. The *sfr8*/*mur1* mutant displays freezing sensitivity that can be rescued by borate	• Panter et al. (2019)
	High temperature	• PME activity is induced; mutants lacking PME34/PME28 are hypersensitive to heat. Guard-cell-expressed PME53 regulates pectin demethylesterification, stomatal function, and guard-cell wall flexibility	• Huang et al. (2017), Wu et al. (2022)
		• Species-specific reduction in pectin (coffee) or arabinose/galactose (tomato)	• Mitcham and McDonald (1992), Lima et al. (2013)
	High osmolarity/drought	• Pectinase application increases hydrotropic responses. The *fei1 fei2 arh1* triple mutant shows exaggerated hydrotropic bending and osmotic hypersensitivity	• Chang et al. (2024)
		• Drought-tolerant maize displays enhanced RG-I arabinan substitution and drought-induced HG remodeling	• Calderone et al. (2024)
		• Soybean drought-tolerant genotypes upregulate *RGXT1–3* (RG-II modification)	• Coutinho et al. (2021)
	Salinity	• PME activity is induced, and HG shows reduced methylesterification. *fer* mutants are highly salt sensitive and can be rescued by pectin crosslinkers	• Feng et al. (2018), Gigli-Bisceglia et al. (2022)
	Pathogens	• PME activity increases during pathogen infection (e.g., *Pseudomonas syringae* in *Arabidopsis*); PME inhibitors enhance defense capacity across species (e.g., *PMEI3* in common bean; *CaPMEI1* in pepper)	• An et al. (2008), Bethke et al. (2014), De la Rubia et al. (2024)
		• HG methylesterification is critical for fungal resistance. PMEI-mediated HG demethylation enhances defense (*Arabidopsis* and cotton). In *Arabidopsis*, the receptor RFO1 is required to sense demethylated HG during *Fusarium* infection	• Lionetti et al. (2007), Lionetti et al. (2017), Liu et al. (2018), Huerta et al. (2023)
		• RG-I arabinose is redistributed to AGPs during *P*. *syringae* infection	• Kim et al. (2023)
		• Reduced pectin content or acetylation limits biotrophic infection: *Arabidopsis pmr5* and *pmr6* restrict biotrophic pathogens; altered pectin acetylation also impacts immunity in maize	• Vogel et al. (2002), Vogel et al. (2004), Engelsdorf et al. (2017), Chiniquy et al. (2019)
		• PGIPs limit pathogen PG activity and enhance OG formation in common bean and *Arabidopsis*	• Federici et al. (2006), Xiao et al. (2024)
Cellulose	High temperature	• Accelerated CSC movement but reduced crystalline cellulose. The *rsw1*/*cesa1* mutant shows radial swelling due to CSC instability	• Fujita et al. (2013), Williamson et al. (2001)
		• *rsw2* mutant (a temperature-sensitive hypomorphic KOR1 allele) shows radial swelling at the restrictive temperature	• Lane et al. (2001), Mansoori et al. (2014), Vain et al. (2014)
	High osmolarity/drought	• Osmotic perturbation redistributes CESAs into SmaCCs/MASCs, transiently reducing cellulose deposition; recovery requires CSI1	• Crowell et al. (2009), Gutierrez et al. (2009), Endler et al. (2015)
		• *sos6-1* (CSLD5 defect) is hypersensitive to osmotic stress	• Zhu et al. (2010)
	Salinity	• Crystalline cellulose is unchanged in the wild type under salt stress; however, cellulose biosynthesis is impaired in *cc1 cc2*	• Kesten et al. (2019), Zhang et al. (2024), Zou et al. (2024)
		• FER phosphorylates CC1/CC2 to stabilize CSCs	• Liu et al., 2024c
		• FER, MIK2, and THE1 link salt stress sensing to the inhibition of cellulose biosynthesis	• Van der Does et al. (2017), Yang et al. (2023), Zhai et al. (2024)
	Pathogens	• *Xanthomonas* uses the T2SS to secrete cellulases, promoting vascular colonization	• Szczesny et al. (2010), Chang et al. (2014)
		• *Ralstonia solanacearum* targets CSCs at lateral root emergence sites	• Yu et al. (2024)
Hemicelluloses and lignin	High osmolarity/drought	• Drought suppresses *XYLANASE1* (*XYN1*), leading to alterations in the xylem cell wall. Enhanced drought resistance of *xyn1* is suppressed by the *cle26* mutation	• Endo and Fukuda (2024)
		• Drought-tolerant maize lines display higher *p*-coumarate levels associated with wall remodeling	• Calderone et al. (2024)
		• Drought-tolerant triticale cultivars accumulate higher levels of cell wall-bound phenolics, primarily ferulic acid	• Hura et al. (2012)
	Pathogens	• *Xanthomonas* T2SS secretes xylanases that degrade hemicellulose, facilitating infection in *Arabidopsis* and rice	• Szczesny et al. (2010), Chang et al. (2014)
		• FER regulates stress-induced lignification: in *Arabidopsis*, FER promotes RD26 degradation during *Ralstonia solanacearum* infection; in tomato, the RALF2–FER–MYB63 module drives lignification during *Fusarium* infection	• Liu et al. (2023), Fan et al. (2024), Wang et al. (2024a, 2024b)
Glycoproteins	Salinity	• AGPs increase under salinity in *Arabidopsis*, and mutants impaired in Hyp-O-galactosylation show salt hypersensitivity. AGP application restores growth in *murus4*. Seagrasses incorporate glucuronic acid into AGPs to bind Ca^2+^ and stabilize cell walls	• Lahaye and Epstein (1969), Lamport et al. (2006), Basu et al. (2015), Pfeifer and Classen (2020), Pfeifer et al. (2020)
		• Salt stress increases extensin levels; arabinosylation is required to control wall porosity and modulate root bending under saline conditions. LRXs bind RALF peptides and interact with *Cr*RLK1Ls to maintain cell wall integrity under salinity; loss of LRX3/4/5 causes hypersensitivity	• Zhao et al. (2018, 2021), Zou et al. (2024)
	High osmolarity/drought	• Desiccation-tolerant species (*Mohria caffrorum* and *Myrothamnus flabellifolia*) accumulate arabinose-rich pectins and AGPs	• Moore et al. (2013)
	Pathogens	• Redistribution of arabinose from RG-I to AGPs upon *P*. *syringae* infection enhances resistance	• Kim et al. (2023)

Note: The table summarizes wall modifications reported in the studies cited in this review. Conclusions are based on work carried out in *Arabidopsis thaliana* unless otherwise specified.

### Pectin remodeling: A common theme under stress

#### Temperature-induced pectin modification

Pectins are among the most dynamic and stress-sensitive polysaccharides of the plant cell wall, and their remodeling represents a central adaptive mechanism under both abiotic and biotic stress conditions. In *Arabidopsis*, exposure to low temperatures (<10 °C) reduces cell expansion and growth (Penfield, 2008), whereas high temperatures promote thermonastic elongation of roots, hypocotyls, and petioles to enhance cooling and minimize sun exposure (Hayes, 2019; Vu et al., 2021; Quint et al., 2023). It has been proposed that the cell wall contributes to freezing tolerance by acting as a barrier that prevents plasma membrane crystallization and restricts ice formation (Takahashi et al., 2021). Conversely, heat stress promotes primary wall elongation while suppressing secondary wall thickening, thereby favoring flexibility over rigidity (Wei et al., 2023). Freezing induces characteristic modifications in pectic polysaccharides. β-1,4-galactans decorating rhamnogalacturonan I (RG-I) side chains accumulate under freezing stress, and mutations in *GALACTAN SYNTHASE* genes (*gals1 gals2 gals3*) impair freezing tolerance (Takahashi et al., 2024). Similarly, arabinose-rich RG-I side chains also accumulate under cold stress (Takahashi et al., 2019, 2021). By contrast, heat stress induces species-specific and sometimes contradictory modifications: tomato shows reduced wall galactose and arabinose content, whereas coffee exhibits decreased pectin levels together with increased water-soluble arabinogalactans (Mitcham and McDonald, 1992; Lima et al., 2013). These discrepancies complicate the formulation of a unified model of RG-I function under thermal stress, particularly given the proposed role of galactans in cold-induced wall rigidification (Takahashi et al., 2019). Freezing tolerance also depends on RG-II. A genetic screen identified SENSITIVE TO FREEZING 8 (SFR8/MURUS1), which encodes GDP-D-MANNOSE-4,6-DEHYDRATASE, a fucose biosynthetic enzyme in *Arabidopsis* (Bonin et al., 1997; Panter et al., 2019). *sfr8*/*mur1* mutants exhibit reduced wall fucose content and impaired RG-II dimerization, resulting in freezing sensitivity that can be alleviated by borate application, which enhances RG-II crosslinking via apiose–apiose bonds (Panter et al., 2019). These findings highlight the importance of cell wall strengthening and crosslinking for tolerance to low temperatures. Cold stress induces PME activity in a BRASSINOSTEROID INSENSITIVE 1 (BRI1)-dependent manner, with PME41 proposed as a key contributor to brassinosteroid-mediated wall remodeling during cold stress in *Arabidopsis* (Qu et al., 2011). Overexpression of *CbPMEI1* from *Chorispora bungeana* or its *Arabidopsis* homolog *PMEI13* renders plants freezing-sensitive but promotes longer root growth under cold conditions (10 °C), indicating finely tuned, temperature-dependent regulation of PME activity (Chen et al., 2018). Heat stress also induces PME activity (Huang et al., 2017), and *Arabidopsis* mutants lacking PME34 and PME28 are hypersensitive to high temperatures (Huang et al., 2017). In addition, the guard-cell-specific PME53 contributes to thermotolerance: the *pme53* mutant displays altered PME activity, impaired abscisic acid (ABA)-induced stomatal closure, and reduced heat tolerance, consistent with a role for PME53-mediated HG demethylesterification in maintaining guard-cell wall flexibility under heat stress (Wu et al., 2022). While HG demethylation under heat stress can activate PGs and promote wall loosening (Höfte et al., 2012), how PME-driven HG modifications give rise to opposite wall behaviors under cold versus heat stress remains unresolved.

#### Integration of SSPs and pectin dynamics under water stress

Pectin modification is also affected under stress conditions related to water availability and distribution. For example, drought and osmotic stress alter the hydration state of the cell wall matrix, which can disrupt polymer organization and strengthen hydrogen bonding—mechanisms that physical studies and recent analyses suggest contribute to reduced wall extensibility (Tako, 2015; John et al., 2022; Etale et al., 2023). Osmotic treatments (e.g. mannitol, sorbitol, or polyethylene glycol) are commonly used in laboratory settings to mimic these effects and trigger hydrotropism, a plant response that redirects growth toward regions of higher water potential (Takano et al., 1995). The RLKs FEI1, FEI2, and ALTERED ROOT HYDROTROPIC RESPONSE 1 (ARH1) have been identified as regulators of hydrotropism (Chang et al., 2024). Overexpression of these genes enhances osmotic tolerance, whereas *fei1 fei2 arh1* triple mutants exhibit exaggerated root bending under osmotic stress. Notably, pectinase application further increases bending and osmotic sensitivity (Chang et al., 2024), indicating a strong functional link between hydrotropism and pectin integrity. Osmotic stress induces the expression of four different SSPs belonging to the PSK precursor family (*ProPSK1*, *ProPSK3*, *ProPSK4*, and *ProPSK5*), as well as several SUBTILISIN-LIKE PROTEASEs (*SBT1*.*4*, *SBT3*.*7*, and *SBT3*.*8*), which are implicated in both peptide maturation and PME activity (Stührwohldt et al., 2021). *sbt* mutants, such as *sbt3*.*8*, are hypersensitive to osmotic stress and display enhanced growth inhibition that can be rescued by PSK treatment, suggesting that subtilases are required for the activation and maturation of these signaling peptides. Consistent with this interpretation, overexpression of *ProPSK1* and *SBT3*.*8* enhances tolerance to water stress (Stührwohldt et al., 2021). PSK treatment (the conserved tyrosylated pentapeptide) has also been shown to alter cell wall composition in carrot protoplasts (Godel-Jędrychowska et al., 2019). However, it remains unclear whether PSK signaling can counteract drought-induced wall modifications *in planta* and how PSK signaling is mechanistically linked to pectin remodeling. Studies in crop species indicate that drought tolerance is frequently associated with pectin remodeling. Drought-tolerant wheat lines exhibit increased RG-I and RG-II side chains under stress, likely enhancing cell wall hydration capacity (Leucci et al., 2008). Similarly, tolerant maize lines display enhanced RG-I arabinan substitution, but also higher *p*-coumarate levels, and drought-induced HG remodeling (Hura et al., 2012; Calderone et al., 2024). Modifications in cell wall-localized glycoproteins—which often link pectic saccharides and contribute to their stability—have also been observed in the desiccation-tolerant species *Mohria caffrorum* and *Myrothamnus flabellifolia*. These species accumulate arabinose-rich pectins and AGPs, possibly as a strategy to facilitate rapid rehydration after prolonged desiccation (Moore et al., 2013). In soybean, drought-tolerant genotypes upregulate homologs of *Arabidopsis RG*-*XYLOSYLTRANSFERASE* genes (*RGXT1, RGXT2,* and *RGXT3*), which catalyze the transfer of a xylose residue onto fucose in RG-II side chains—a modification essential for pectin structure and function (Egelund et al., 2006, 2008; Liu et al., 2011; Coutinho et al., 2021). Members of the RALF peptide family have also been linked to drought sensitivity. Among them, overexpression of *RALF8* increases drought sensitivity in *Arabidopsis*, resulting in stunted root systems with long and abundant root hairs (Atkinson et al., 2013). FER is required for RALF8-induced root growth inhibition (Frederick et al., 2019; Abarca et al., 2021). Because *fer* mutants show reduced sensitivity to mannitol (Chen et al., 2016; Feng et al., 2018), it is possible that FER–RALF8 signaling alters root morphology in ways that increase susceptibility to water stress.

#### SSPs and pectin are regulators of salinity tolerance

Responses to osmotic and salt stresses share similarities because salinity imposes both osmotic and ionic stress (Yang and Guo, 2018). However, these stresses are not identical. For example, *Arabidopsis fer-4* mutants, which are resistant to osmotic stress, are highly susceptible to sodium chloride (hereafter referred to as salt) (Chen et al., 2016; Feng et al., 2018). In addition to imposing osmotic stress, salinity alters pectin structure. Specifically, salt stress triggers PME activity and subsequent pectin demethylesterification. Demethylesterified pectin, which accumulates under salinity (Gigli-Bisceglia et al., 2022), can bind and activate FER (Feng et al., 2018; Lin et al., 2022), thereby triggering signaling pathways that help maintain CWI under salt stress. The pronounced salt sensitivity of *fer-4* mutants can be alleviated by application of HG and RG-II crosslinkers (Feng et al., 2018). Because FER preferentially binds demethylesterified pectin (Feng et al., 2018; Lin et al., 2022), it likely plays a direct role in sensing or stabilizing salinity-induced wall modifications. Several RALF peptides are also associated with salinity responses. Loss of FER or of LRX3/4/5 leads to hypersensitivity to salt stress, a phenotype that can be phenocopied by overexpression of *RALF22 or RALF23* (Zhao et al., 2018). Application of RALF22/23 promotes FER internalization, suggesting that RALF perception inhibits FER function and creates a FER loss-of-function-like state (Zhao et al., 2018). In wild-type plants, salt stress promotes maturation of RALF22 via the protease SBT6.1/S1P (Srivastava et al., 2009; Zhao et al., 2018). Why salt-induced RALF22 maturation does not result in the same extreme hypersensitivity observed in *fer* or *lrx3*/*4*/*5* mutants remains unresolved. RALF1 signaling also intersects with salinity responses. *ProRALF1* expression is suppressed under salt stress, yet exogenous RALF1 exacerbates leaf bleaching and Na^+^ accumulation (Yu and Assmann, 2018). RALF1 activity depends on the presence of demethylesterified pectin, as inhibition of PME activity suppresses RALF1-mediated growth inhibition (Rößling et al., 2024). Similar to RALF22, RALF1 binds demethylesterified pectin (Rößling et al., 2024). Why RALF1, RALF22, and RALF23 promote salt hypersensitivity rather than protection remains unclear, pointing to a more complex regulatory mechanism. One possibility is that excessive RALF signaling interferes with FER–pectin interactions, thereby inhibiting FER function and preventing the receptor from sensing protective pectin-derived cues, ultimately leading to stress overactivation. Recent work suggests that this paradox may be explained by pectin–RALF-driven phase separation at the cell wall–plasma membrane interface, which clusters FER–LLG1 and other regulators into condensates, amplifying stress responses and promoting receptor endocytosis (Liu et al., 2024a). Although FER modulates PME activity, treatment with RALF23 reduces PME activity even in *fer* knockout mutants (Biermann et al., 2025), suggesting that PME responsiveness is regulated by RALF peptides, which may act both as structural components of the cell wall and as signaling molecules.

Some of the pathways controlling osmotic stress tolerance overlap with salt stress responses, reflecting the dual nature of salinity as both an osmotic and an ionic stress. For example, the SOS5/FLA4–FEI1/FEI2 module, which regulates hydrotropism and pectin-dependent cell wall integrity under osmotic stress, also plays a central role in salinity tolerance. SOS5/FLA4 is required for maintaining cell–cell adhesion and proper root growth under salt stress (Shi et al., 2003). Consistently, mutants of Hyp-*O*-GALACTOSYLTRANSFERASEs, which initiate AGP glycosylation, exhibit salt hypersensitivity phenotypes similar to those of *sos5*/*fla4* mutants (Basu et al., 2015), reinforcing the importance of correct AGP glycosylation for salt resilience. Indeed, AGP levels increase under salt stress (Lamport et al., 2006), and exogenous application of AGP-rich gum arabic rescues the salt-induced root growth defects in *murus4* mutants, which are defective in UDP-D-XYLOSE 4-EPIMERASE 1 and consequently show reduced L-arabinose content (Burget and Reiter, 1999; Burget et al., 2003; Zhao et al., 2019). Seagrasses incorporat negatively charged glucuronic acid into AGPs to preferentially bind calcium, thereby stabilizing their walls under saline conditions (Lahaye and Epstein, 1969; Pfeifer and Classen, 2020; Pfeifer et al., 2020). SOS5/FLA4 interacts with FEI1/FEI2 to form a signaling pathway that regulates levels of the ethylene precursor 1-aminocyclopropane-1-carboxylate (ACC) under both salt and osmotic stress (Seifert, 2021). The swollen root phenotype of *sos5*/*fla4* and *fei1*/*fei2* mutants can be suppressed by inhibiting ethylene biosynthesis, linking this module to cell wall remodeling through ethylene homeostasis (Basu et al., 2016). Ethylene accumulation antagonizes ABA biosynthesis, whereas exogenous ABA partially or fully suppresses both the salt-induced phenotypes of *sos5*/*fla4* mutants (Seifert et al., 2014) and the salt-triggered root cell death in *fer-4* (Lamers et al., 2025). The involvement of ABA in cell wall regulation is further supported by the observation that ABA signaling mutants develop thinner secondary walls (Liu et al., 2021) and that ABA modulates xylem developmental plasticity under water-limiting conditions (Ramachandran et al., 2021). In addition, the putative cell wall integrity sensor THE1 negatively regulates ABA biosynthesis and cell wall mechanics (Bacete et al., 2022), linking ABA signaling to both wall integrity and mechanical properties. Interestingly, although *the1-1* responds normally to salt stress, it alleviates the salt sensitivity of *mik2* loss-of-function mutants (Van der Does et al., 2017). Moreover, mutations in eight members of the SBT3 family, which are required for processing SCOOP peptides—the ligands of MIK2—result in salt sensitivity comparable to that of *mik2* mutants (Yang et al., 2023). Notably, SBT3.3 and SBT3.5 also process PMEs (Coculo et al., 2023), indicating that these proteases regulate the maturation not only of SSPs but also of PME enzymes. The fact that SBTs process multiple substrates complicates functional interpretation, as a single protease may regulate distinct signaling pathways. For example, SBT6.1/S1P processes RALFs, GOLVEN1, and the membrane-localized transcription factor bZIP17 (Srivastava et al., 2009; Ghorbani et al., 2016; Cho, 2024). Accordingly, *s1p* mutant roots are hypersensitive to NaCl, KCl, LiCl, and mannitol (Liu et al., 2007); however, it remains unclear whether this salt susceptibility results from altered processing of SSPs or from effects on other substrates, such as PMEs, which are critical for maintaining pectin integrity under stress.

Other cell wall-localized proteins also play important roles in salinity tolerance. For example, combining a gain-of-function allele of *THE1* with loss of function of HERCULES RECEPTOR KINASE 1 (HERK1) increases salt sensitivity (Gigli-Bisceglia et al., 2022), suggesting that additional members of the *Cr*RLK1L family are essential for maintaining CWI under stress. This observation raises the possibility that THE1 and HERK1 stabilize FER, facilitate its interaction with RALFs, or assemble multiprotein complexes critical for downstream signaling. Beyond LRX proteins, other cell wall-localized glycoproteins also appear to be important. Salt stress increases extensin levels, and arabinosylation of extensins is required to regulate wall porosity and modulate root bending under saline conditions (Zou et al., 2024). Together, these findings indicate that glycosylation contributes to salt tolerance not only through chemical interactions, such as ion binding, but also by modifying cell wall properties to reduce rigidity and enhance flexibility.

#### Intersection of pectin remodeling and SSP signaling in immune responses

Under biotic stress, pathogens manipulate pectin remodeling to promote infection, whereas plants actively counteract these modifications to mount effective defenses. Pathogenic bacteria typically colonize the apoplast and gain entry through stomata, wounds, or sites of lateral root emergence (Melotto et al., 2006; Faulkner and Robatzek, 2012). At *Pseudomonas syringae* infection sites, *Arabidopsis* exhibits increased arabinose content and redistribution from RG-I to AGPs, changes that enhance resistance (Kim et al., 2023). Similar cell wall remodeling occurs in bean leaves infected with *P*. *syringae* pv. *phaseolicola* (De la Rubia et al., 2024). PME activity increases during infection by *P*. *syringae* pv. *maculicola*, and several PMEs contribute to resistance (Bethke et al., 2014). Conversely, loss of PME inhibitors compromises defense against *Pseudomonas* and *Xanthomonas* species in both *Arabidopsis* and pepper (An et al., 2008; De la Rubia et al., 2024), underscoring the importance of tightly regulated HG methylesterification for immunity. Filamentous pathogens, including fungi and oomycetes, invade host tissues either through natural openings or by directly penetrating epidermal cell walls, a process facilitated by the secretion of cell wall-degrading enzymes (O’Connell et al., 2012; Kubicek et al., 2014). In *Arabidopsis powdery mildew resistant 5* (*pmr5) and pmr6 mutants,* reduced pectin content and altered pectin acetylation restricts the proliferation of biotrophic hyphae following infection with *Erysiphe cichoracearum* or *Colletotrichum higginsianum* (Vogel et al., 2002, 2004; Engelsdorf et al., 2017; Chiniquy et al., 2019). More broadly, pectin methylation status is a critical determinant of resistance against fungal pathogens such as *Botrytis cinerea*, *Verticillium dahliae*, and *Fusarium oxysporum* (*F. oxysporum;* Lionetti et al., 2007, 2017; Liu et al., 2018; Huerta et al., 2023). During *F. oxysporum* 5176 infection, reduced HG methylation is perceived by RESISTANCE TO FUSARIUM OXYSPORUM 1 (RFO1), which triggers downstream defense responses (Huerta et al., 2023). Conversely, several pathogens, including *Botrytis* *cinerea*, *Fusarium graminearum*, and *Phytophthora sojae*, rely on PME expression to facilitate PG-mediated degradation of host pectin (Valette-Collet et al., 2003; Sella et al., 2016; Xia et al., 2024). During *P. sojae* infection, soybean induces expression of the PME inhibitor *GmPMI1*, thereby counteracting pectin degradation (Xia et al., 2024). In addition, host-derived PG-INHIBITING PROTEINs (PGIPs) directly restrict pathogen PG activity while promoting the accumulation of elicitor-active pectin fragments, known as oligogalacturonides (OGs) (Federici et al., 2006; Xiao et al., 2024). For example, bean (*Phaseolus vulgaris*) *Pv*PGIP2 interacts with *Fusarium phyllophilum* PG to favor the production of long-chain OGs while limiting shorter, immunosuppressive fragments (Xiao et al., 2024).

Interaction of bacteria or filamentous pathogens with their host plants involves peptide signaling pathways that link immune responses to cell wall remodeling. For example, pathogen-associated molecular pattern (PAMP)-induced secreted peptides (PIPs) are induced in *Arabidopsis* upon pathogen infection and enhance resistance to both bacterial and fungal pathogens (Hou et al., 2014; Yu et al., 2023). PIP1 suppresses the expression of genes associated with pectin catabolism, thereby connecting PIP-induced immunity to cell wall remodeling (Yu et al., 2023). *F*. *oxysporum* exploits the PSY1 and PSK signaling systems to enhance virulence (Shen and Diener, 2013). Similarly, loss of PSY and PSK receptors enhances resistance to *P*. *syringae*, whereas resistance to the necrotrophic fungus *Alternaria brassicicola* is compromised in these receptor mutants (Igarashi et al., 2012; Mosher et al., 2013). PSY1 acts through its receptor PSY1R to regulate cell expansion (Kaufmann and Sauter, 2019). Consistent with this function, genes involved in cell wall modification are induced in response to PSY1 application, potentially supporting cell wall loosening (Mahmood et al., 2014). Recent work has shown that perception of PSY-family peptides by PSYRs 1–3 balances growth with stress responses. Notably, these receptors activate stress-related transcription factors when ligands are depleted, whereas peptide binding promotes growth by repressing stress signaling. This ligand-deprivation mechanism highlights the role of PSY–PSYR signaling in coordinating the trade-off between growth and defense under fluctuating environments (Ogawa-Ohnishi et al., 2022). Homologs of RALF peptides have been identified in multiple phytopathogenic fungi, some of which elicit RALF-like responses in plants or promote infection success through interaction with FER (Masachis et al., 2016; Thynne et al., 2017). For example, a *F*. *graminearum* RALF peptide induces apoplastic alkalinization and growth suppression in wheat, tomato, and *Arabidopsis* and requires interaction with FER to suppress PTI responses (Wang et al., 2024a). FER also forms a RALF-regulated scaffold with pattern-recognition receptors, thereby influencing immune signaling and linking CWI to immunity (Stegmann et al., 2017; Malivert and Hamant, 2023). Nevertheless, some immunity-related functions of host-encoded RALFs appear to be FER independent and may involve direct RALF–cell wall interactions (Leicher et al., 2025).

### Stress on the scaffold: Alterations of load-bearing cell wall elements are connected with SSP signaling

In addition to pectin modifications that enable rapid changes in cell wall properties, both abiotic and biotic stresses exert profound effects on cellulose biosynthesis and the organization of cellulose microfibrils. Exposure to elevated temperatures accelerates the movement of CSCs at the plasma membrane while concomitantly reducing crystalline cellulose content. This observation suggests that, although cellulose synthesis is enhanced, the resulting microfibrils may be less organized (Fujita et al., 2013). Mutants defective in cellulose synthesis further highlight this temperature sensitivity. The *radial swollen 1* (*rsw1*/*cesa1*) mutant exhibits heat-induced radial swelling caused by impaired CSC stability and reduced cellulose crystallinity (Williamson et al., 2001; Fujita et al., 2013; Kumar and Turner, 2015). Similarly, the *rsw2* mutant, which carries a point mutation in the endo-1,4-β-D-glucanase KORRIGAN 1—a protein that facilitates cellulose production by trimming glucan chains and supporting proper microfibril assembly—also displays temperature-sensitive defects (Baskin et al., 1992; Lane et al., 2001; Mansoori et al., 2014; Vain et al., 2014). Together, these findings indicate that intact CSC machinery, proper microfibril assembly, and coordinated carbohydrate–carbohydrate interactions are essential for preserving cell wall architecture and enabling normal cell expansion under high-temperature stress. In contrast, the effects of low temperatures on cellulose remain largely unexplored, as no clear alterations in cellulose content, crystallinity, or CSC dynamics have yet been reported.

Several EPFL-family peptides act as ligands for the ERECTA receptor kinase to regulate stamen filament and pistil growth (Kawamoto et al., 2020; He et al., 2023). At cool temperatures (16 °C), EPFL6 is required to promote filament elongation by driving cell proliferation, thereby enabling stamens to reach the pistil (Negoro et al., 2023). In addition to its developmental roles, ERECTA has also been implicated in cell wall-mediated immunity and the regulation of CWI (Sánchez-Rodríguez et al., 2009; Bacete et al., 2018). It therefore remains to be determined whether EPFL6–ERECTA signaling integrates temperature sensing with wall-related defense pathways. The expression of the CLE45 peptide in pistils is spatially regulated in a temperature-dependent manner to maintain pollen-tube reception under high-temperature conditions (Endo et al., 2013). CLE45 is also expressed in vascular tissue, and its transport destination depends on the cell wall structure of the xylem (Endo et al., 2019). These findings led to the proposal that stress signaling influences xylem transport of CLE peptides through the induction of cell wall modification (Endo et al., 2019). During drought stress, CLE25 is expressed in roots and transported to the shoot, where it induces ABA accumulation and triggers stomatal closure to reduce water loss (Takahashi et al., 2018; Bharath et al., 2021). By contrast, CLE26 does not contribute to immediate drought resistance but instead functions in a cell wall-dependent drought stress memory mechanism. Short-term dehydration suppresses Xylanase 1 (XYN1), leading to changes in xylem cell wall composition and the accumulation of CLE26 in leaves (Endo and Fukuda, 2024). Notably, enhanced drought resistance in *xyn1* mutants is suppressed in the *xyn1 cle26* double mutant, supporting the conclusion that xylem cell wall modifications are required to activate CLE26 signaling and promote resistance to repeated drought stress (Endo and Fukuda, 2024).

In *Arabidopsis*, osmotic perturbation causes the redistribution of CESAs into small CESA compartments (SmaCCs) or microtubule-associated CESA compartments (MASCs), resulting in a transient reduction in cellulose deposition (Crowell et al., 2009; Gutierrez et al., 2009; Endler et al., 2015). Under stress conditions, these compartments show enhanced association with cortical microtubules, and recovery of cellulose synthesis requires CELLULOSE SYNTHASE INTERACTIVE 1 (CSI1) (Lei et al., 2015), which tethers CSCs to microtubules and facilitates their recycling back to the plasma membrane (Endler et al., 2015). Although crystalline cellulose levels remain unchanged in wild-type *Arabidopsis* seedlings exposed to salt (Zhang et al., 2023; Zou et al., 2024), cellulose biosynthesis is severely compromised in the *cellulose synthase companion protein 1* and *2* double mutant (*cc1 cc2*) (Kesten et al., 2019). Upon salt stress, these mutants display reduced crystalline cellulose content and defective recovery of cortical microtubules, demonstrating that COMPANION OF CELLULOSE SYNTHASE proteins (CC1 and CC2) are required to maintain microtubule dynamics and the plasma membrane localization of CSCs under salinity stress (Endler et al., 2015; Kesten et al., 2019). Genetic studies reinforce the importance of cellulose-related pathways in osmotic tolerance. For example, the *salt overly sensitive 6-1* (*sos6-1*) mutant, which is defective in CELLULOSE SYNTHASE-LIKE D5 (CSLD5), exhibits hypersensitivity to osmotic stress (Zhu et al., 2010). Mechanistically, the receptor-like kinase FER has been shown to phosphorylate CC1 and CC2, thereby stabilizing cortical microtubule arrays during salt stress and linking CWI sensing to CESA activity (Liu et al., 2024c). Another connection between salt stress and cellulose biosynthesis is provided by the SCOOP receptor MIK2 and the *Cr*RLK1L THE1. Both receptors are required for activation of CWI signaling in response to impaired cellulose biosynthesis, and MIK2-mediated promotion of salt-stress tolerance depends on THE1 (Julkowska et al., 2016; Van der Does et al., 2017; Zhai et al., 2024).

Biotic stresses also target cell wall remodeling, with profound consequences for plant immunity and pathogen virulence. Consistent with this view, alterations in cell wall composition have been linked to distinct resistance phenotypes against pathogens with different lifestyles (Molina et al., 2021). Bacterial pathogens frequently exploit secretion systems to compromise host cell walls. In Xanthomonas, the type II secretion system (T2SS) releases cell wall-degrading enzymes (CWDEs), including cellulases and xylanases, which not only promote virulence but also facilitate type III secretion system (T3SS)-dependent effector delivery (Szczesny et al., 2010; Chang et al., 2014; Solé et al., 2015). *Xanthomonas campestris* pv. *campestris* (*Xcc*) requires a functional T2SS to colonize the vasculature after entering *Arabidopsis* leaves through hydathodes, and functional genomics has identified four conserved CWDEs in *Xcc* that contribute non-redundantly to T2SS-dependent vascular spread (Paauw et al., 2024). *Ralstonia solanacearum* provides a more direct example of interference with cellulose biosynthesis, as it targets the CESA–CSI1 interaction at sites of lateral root emergence to promote infection (Yu et al., 2024). Chemical inhibition of cellulose synthesis with isoxaben phenocopies this effect, leading to increased lateral root formation and enhanced susceptibility to *R*. *solanacearum* (Yu et al., 2024). However, cellulose deficiency can also activate defense pathways, as it triggers jasmonic acid–dependent immune signaling (Desaint et al., 2024). Xylem lignification restricts *R*. *solanacearum* proliferation in *Arabidopsis*, tomato, and tobacco and is negatively regulated by FER (Wang et al., 2024b). Given recent evidence that FER senses alterations associated with changes in lignin composition, it is plausible that increased lignification upon pathogen infection is perceived through FER-mediated pathways, which may subsequently restrain further lignin deposition to balance defense and growth (Liu et al., 2023; Wang et al., 2024b). Supporting this model, lignin deposition in tomato during infection by *F*. *oxysporum* f. sp. *lycopersici* is regulated by a RALF2–FER–MYB63 signaling module that fine-tunes defense activation (Fan et al., 2024). During *F*. *oxysporum* infection of *Arabidopsis*, downregulation of primary cell wall cellulose synthesis genes precedes the induction of canonical defense-related genes, including *ProPEP3* and *PEPR1*, and cellulose deficiency can promote defense activation through ethylene signaling (Menna et al., 2021).

## Transcriptional regulation of SSP precursors under cell wall-related stress

Current knowledge, as outlined above, illustrates how SSP signaling and cell wall remodeling intersect during stress exposure. Members of the same SSP families can be involved in responses to different stress conditions. For example, substantial overlap exists between peptide-mediated regulation of pattern-triggered immunity (PTI) and salt stress responses (Gigli-Bisceglia and Testerink, 2021). Peptides from the PEP family and CAP superfamily–derived peptides (CAPEs), both well-established regulators of pathogen responses, have also been implicated in salinity stress, albeit with opposing effects (Chien et al., 2015; Nakaminami et al., 2018; Saijo et al., 2021). *CAPE1* is transcriptionally downregulated under high salinity and negatively regulates plant salt tolerance by repressing salt-tolerance genes (Chien et al., 2015). By contrast, *ProPEP* genes are strongly upregulated under salt stress (Nakaminami et al., 2018; Gigli-Bisceglia et al., 2022). PEPs appear to function as negative regulators of stress responses under conditions of cell wall damage and salinity, as exogenous application of PEPs or overexpression of *ProPEP*s alleviates phenotypes caused by salt stress and inhibition of cellulose biosynthesis (Engelsdorf et al., 2018; Nakaminami et al., 2018; Morton et al., 2025; Zhang et al., 2025b). The attenuation of *ProPEP3* expression by calcium treatment suggests that pectin status modulates PEP signaling under salt stress (Gigli-Bisceglia et al., 2022), or alternatively, that PEP signaling is sensitive to cell wall modifications. How PEPs—well-established positive regulators of PTI (Bartels and Boller, 2015)—exert opposite effects under conditions of cell wall damage remains unresolved.

To explore how publicly available transcriptomic resources can inform hypotheses about SSP function under stress, we reanalyzed RNA-sequencing datasets from *Arabidopsis*. Our analysis focused on SSP precursor transcripts, as their transcriptional regulation often reflects SSP activity in defined stress contexts (Engelsdorf et al., 2018; Nakaminami et al., 2018; Gigli-Bisceglia et al., 2022; Chen et al., 2023; Zhai et al., 2024). We examined a broad range of abiotic stresses, including cold, heat, drought, salt, and osmotic stress induced by sorbitol (Hanada et al., 2013; Bacete et al., 2022); biotic stresses, including infection by *Hyaloperonospora arabidopsidis* isolates Emoy2 and Waco9, *Fusarium oxysporum*, *Botrytis cinerea*, and *Pseudomonas syringae* (Asai et al., 2014; Lewis et al., 2015; Haller et al., 2020; Menna et al., 2021); and direct cell wall perturbations, including isoxaben (ISX) treatment (inhibiting cellulose biosynthesis), *ixr1-1* (a *CESA3* mutant), and PMEI overexpression (PMEIox), which reduces PME activity (Wolf et al., 2012, 2014; Engelsdorf et al., 2018; Zhai et al., 2024). By including wall-targeting conditions, we aimed to assess whether clusters of stress-responsive peptides correlate with known wall-remodeling events. Because these datasets derive from different tissues and developmental stages, the patterns described below should be considered illustrative. Our aim is to highlight possible points of convergence between stress-induced cell wall changes and peptide signaling that may guide future functional validation.

### Transcriptional patterns might reveal candidate SSPs broadly induced under cell wall-related stress

**Figure 3:**
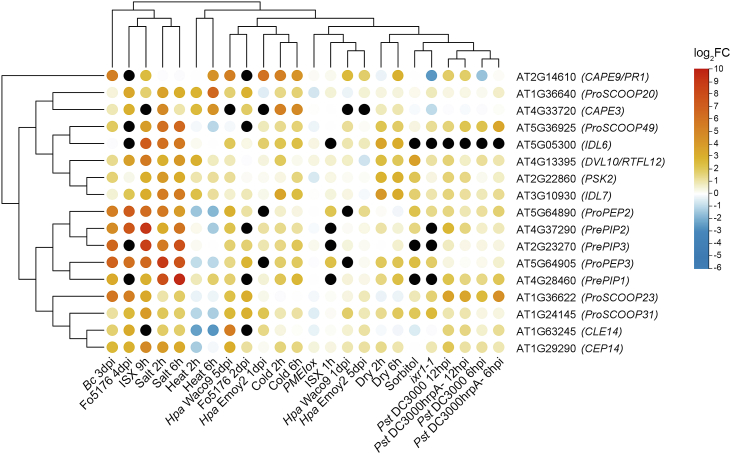
A cluster of precursor peptide transcripts shows increased expression under stress. Global clustering of published transcriptomics datasets identified three main expression clusters (Supplemental Figure 1). Cluster I was extracted and re-clustered to further resolve expression patterns of peptide precursor–encoding genes under abiotic stress, biotic stress, and cell wall-modifying conditions. Two-dimensional hierarchical clustering was performed on both peptide-encoding genes (rows) and experimental conditions (columns) using the Euclidean distance metric and the complete linkage method (Chen et al., 2020). Each circle represents the log_2_ fold change (log_2_FC) in transcript abundance relative to the corresponding control conditions. Colors indicate downregulation (blue), no change (white), and upregulation (red), whereas black circles denote “not available” values corresponding to missing data.

Two-dimensional hierarchical clustering of 162 SSP precursor transcripts (Supplemental Figure 1) grouped stress responses into two main clusters: (A) salt, isoxaben, and *Fusarium* infection, which showed similar trends, consistent with previously proposed overlapping pathways (Gigli-Bisceglia and Testerink, 2021); and (B) a heterogeneous set of biotic and abiotic stresses. Transcript-centered clustering further separated SSPs into three major expression groups, designated cluster I, cluster II, and cluster III (Supplemental Figure 1). Cluster I was characterized by a general upregulation across stress conditions, including wall-targeting treatments (Figure 3). This cluster includes *ProPEP2* and *ProPEP3*, which have been reported to function in abiotic stress, immunity, and cell wall-damage responses (Bartels and Boller, 2015; Engelsdorf et al., 2018; Nakaminami et al., 2018; Saijo et al., 2021; Gigli-Bisceglia et al., 2022), together with *PrePIP1*, *PrePIP2*, and *PrePIP3*, which are mainly linked to PTI (Hou et al., 2014; Najafi et al., 2020; Yu et al., 2023). Additional members include *ProSCOOP23*, *ProSCOOP31*, *CLE14*, and *CEP14*, the latter of which is induced by salicylic acid and perceived by CEPR2–BAK1/SERK4 receptor complexes to trigger immune responses and enhance resistance to *P*. *syringae* (Wang et al., 2024c). These SSP transcripts show broad induction under drought, salinity, pathogen attack, and treatments that compromise wall integrity, while being repressed under heat stress. This pattern suggests that stresses altering wall hydration or pectin composition may influence the expression of peptides involved in CWI signaling. A second subgroup within cluster I comprises *ProSCOOP20*, *ProSCOOP49*, *IDL6*, *IDL7*, *DVL10*/*RTFL12*, *PSK2*, *CAPE3*, and *CAPE9*, the latter derived from the PTI marker gene *PATHOGENESIS-RELATED PROTEIN 1* (*PR1*) (Chen et al., 2023). This subgroup displays consistent upregulation across nearly all stress conditions analyzed, suggesting a broad activation pattern that is largely independent of stress type. While these associations remain correlative, the clustering pattern indicates that wall-related stresses coincide with the induction of distinct signaling peptides rather than the activation of entire SSP families.

### Stress-specific gene-induction patterns indicate overlaps between SSPs of unrelated families

Whereas cluster I may highlight SSPs that are transcriptionally induced under cell wall-related stresses, other clusters display more stress-specific transcriptional regulation, revealing overlaps between otherwise unrelated SSP families. Cluster II (Supplemental Figure 1) displays heterogeneous transcriptional patterns, with SSPs showing both up- and downregulation depending on the type of stress, while also revealing similarities among subsets of SSP expression profiles. Like cluster I, this cluster includes representatives of the CLE, CEP, SCOOP, PSK, PEP, DVL/RTFL, and CAPE families, further emphasizing that transcripts from the same SSP families can follow opposing regulatory trends. Cluster III (Supplemental Figure 1) is characterized by general downregulation across most treatments (e.g., *DVL6*/*RTFL16*, *CLE16*, *CAPE2*, *CLE19*, and *RALF27*), although some genes, such as *DVL16* and *DVL22*, are upregulated during oomycete infections but repressed under cellulose-related and abiotic stresses. By aligning SSP expression clusters with known patterns of cell wall modification under diverse stress conditions, several clear trends emerge. Although these associations are hypothesis-generating rather than mechanistic, they illustrate how mining public transcriptomic data can uncover promising connections between cell wall remodeling and SSP signaling.

## Concluding remarks and perspectives

In this review, we summarize current knowledge of plant cell wall structure and the effects of abiotic and biotic stresses on cell wall remodeling. Given that the cell wall serves as the matrix in which SSPs are released, we first provide an overview of peptide-encoding genes that are either induced by or involved in regulating cell wall-related stress responses. Because current knowledge of SSPs remains limited, we then highlight the use of available transcriptomic datasets, spanning various developmental stages and stress conditions, including those that affect cell wall modifications, to identify common regulatory patterns and stress-specific transcriptional responses. This analysis reveals both common regulatory patterns associated with cell wall stress and distinct, stress-specific transcriptional responses. It further highlights that findings obtained for individual peptides may not necessarily be indicative of the functions of related family members. Although transcriptomic analyses provide a powerful systems-level perspective, they need to be complemented by experimental strategies that directly address how peptides and the cell wall influence each other during stress. Biochemical analyses could clarify whether stress-induced remodeling of wall polymers affects the release, processing, or activity of peptides, for example by testing whether wall-degrading or crosslinking enzymes promote the liberation of peptide precursors or modulate their bioactivity. Genetic approaches, including the combination of peptide mutants with mutants defective in cell wall biosynthesis or remodeling, can help determine whether peptides act upstream as regulators of wall integrity or downstream as effectors of remodeling. Proteomic and peptidomic profiling of apoplastic and wall-associated fractions would further clarify how peptide abundance, localization, and post-translational modifications correlate with stress-induced wall changes, while imaging of fluorescent peptide reporters and super-resolution microscopy could visualize peptide distribution alongside cell wall alterations *in vivo*. These analyses can be reinforced by biophysical approaches, including atomic force microscopy, nanoindentation, and Brillouin microscopy, to directly quantify how the presence or absence of specific peptides influences wall stiffness, porosity, and elasticity under stress conditions. Ultimately, integrating multi-omics and biochemical datasets into network models will enable the identification of regulatory modules in which peptides co-regulate wall-modifying enzymes and wall-integrity sensors, thereby advancing a mechanistic understanding of how peptide signaling and cell wall remodeling are coordinated to shape plant adaptation to abiotic and biotic stresses.

## Funding

This work was supported by the 10.13039/501100001659Deutsche Forschungsgemeinschaft (DFG, German Research Foundation) under grant EN 1071/3-1 to T.E. and by the 10.13039/501100003246Netherlands Organization for Scientific Research (NWO) under grants OCENW.M.24.079 (10.61686/TWDQN34060) and OCENW.XS23.1.050 to N.G-B.

## Acknowledgments

We thank Agnieszka Engelsdorf for assistance with the illustration of the cell wall. No conflict of interest declared.

## Author contributions

T.E. and N.G.-B. conceptualized the review. J.D. performed the transcriptomic analyses. J.D., R.N.M., T.E., and N.G.-B. wrote the manuscript. All authors discussed the content and approved the final version.

## Supplemental information

Document S1. Supplemental Figure 1 and supplemental methods

Supplemental Data 1. Log_2_ fold-change values of peptide precursor-encoding genes across publicly available Arabidopsis stress transcriptomic datasets

Document S2. Article plus supplemental information
