# Supplementary material for: Plant cell wall remodeling and peptide signaling under abiotic and biotic stress
Source: Plant Commun. 2026 Jan 29;7(4):101741. doi: 10.1016/j.xplc.2026.101741 (PMC13084100; doi:10.1016/j.xplc.2026.101741)
Supplement: Document S1. Supplemental Figure 1 and supplemental methods [file mmc1.pdf]

Plant Communications, Volume 7

Supplemental information

Plant cell wall remodeling and peptide signaling under abiotic and biotic stress

Joy Debnath, Richard Noi Morton, Timo Engelsdorf, and Nora Gigli-Bisceglia

Title: Plant Cell Wall Remodeling and Peptide Signaling Under Abiotic and Biotic Stress

Joy Debnath[1], Richard Noi Morton[2], Timo Engelsdorf[2*], Nora Gigli-Bisceglia[1*]

[1] Plant Stress Resilience, Institute of Environmental Biology, Utrecht University, Utrecht, the Netherlands

[2]Molecular Plant Physiology, Department of Biology, Philipps-Universität Marburg, 35043 Marburg, Germany

Supplemental Figure Legends

Figure S1. Two-dimensional hierarchical clustering heatmap of peptide precursor-encoding gene expression under abiotic stress, biotic stress, and cell wall modification conditions. Hierarchical clustering was performed on both peptide-encoding genes (rows) and experimental conditions (columns) using the Euclidean distance metric and the complete linkage method (Chen et al., 2020). The resulting dendrograms illustrate relationships among transcripts and conditions. Three major gene clusters [Cluster I (CI), Cluster II (CII) and Cluster III (CIII)] and two condition groups (A and B) are annotated based on their expression patterns. The color scale represents downregulation (blue), no change (white), and upregulation (red). Black squares indicate "Not Available" (NA) values, corresponding to data that are missing for specific conditions within the datasets. Color coding and handling of missing values are described in *Supplemental methods*.

Supplemental Methods

All transcriptomic datasets analyzed were obtained from publicly available sources in the NCBI Gene Expression Omnibus (GEO) under the following accession numbers: GSE34188, GSE53641, GSE168919, GSE151210, GSE56094, GSE108987, and GSE48596. Cold, heat, drought (Dry) and salt stress responses [from (Hanada et al., 2013)] were used to calculate differential expression at 2-hour and 6-hour treatment time points relative to respective controls using the GEO2R web tool, which employs the limma package for linear modeling and empirical Bayes moderation. For osmotic (sorbitol) stress, log-transformed RPKM values were obtained from (Bacete et al., 2022), and differential expression was assessed in R using the limma package. Transcriptional responses to oomycete (*Hyaloperonospora arabidopsidis*, Hpa) isolates Emoy2 and Waco9 [from (Asai et al., 2014)] were analyzed using raw TPM files, which were merged, log_2-transformed, and analyzed using limma in R. Contrasts were defined as treatment versus water controls at each time point. *Fusarium oxysporum* strain Fo5176 responses were analyzed using raw count data [from (Menna et al., 2021)]. Counts were merged and normalized using edgeR, followed by voom transformation and linear modeling with limma. Contrasts compared infected and mock-treated samples at each time point. *Botrytis cinerea* (*Bc*, 3 days post-inoculation) responses were analyzed from (Haller et al., 2020) using GEO2R (limma), comparing infected and control samples.

Responses to *Pseudomonas syringae* pv. *tomato* DC3000 (*Pst*) wild type and *hrpA-* mutant were analyzed using GEO2R (limma) [from (Lewis et al., 2015)], with each treatment compared to mock controls. Responses to isoxaben (ISX, 1 h and 9 h) and ISX-insensitive mutants (*ixr1-1*) were analyzed using raw count data [from (Engelsdorf et al., 2018; Zhai et al., 2024)]. Counts were merged, normalized with edgeR, and analyzed using the limma-voom pipeline. Contrasts compared ISX-treated and *ixr1-1* samples to DMSO-treated controls. *PMEIox* (pectin methylesterase inhibitor overexpression) responses were analyzed using GEO2R (limma), comparing PMEIox and control samples from (Wolf et al., 2014). Raw and processed data, as well as analysis scripts, are available upon request. Peptide precursor-encoding genes were identified based on *Arabidopsis thaliana* genome annotations and categorized by peptide family. For each gene and condition, $\log_2$ fold change ($\log_2$FC) values were obtained from the corresponding differential expression analyses. Genes lacking data for a given condition are represented in black on the heatmaps. Heatmaps were generated using the Heatmap Illustrator function in TBtools (Chen et al., 2020). The color scale spans from blue (downregulation) to red (upregulation), with white indicating no change in expression and black representing missing values, corresponding to data that were not available (NA) for specific conditions. Missing values can occur for several reasons, including low transcript abundance below detection limits, bioinformatic filtering of low-count or ambiguously mapped reads, or incomplete genome annotations. Hierarchical clustering of both genes and experimental conditions was performed using the Euclidean distance metric and complete linkage method in TBtools (Chen et al., 2020).

Supplemental Figures

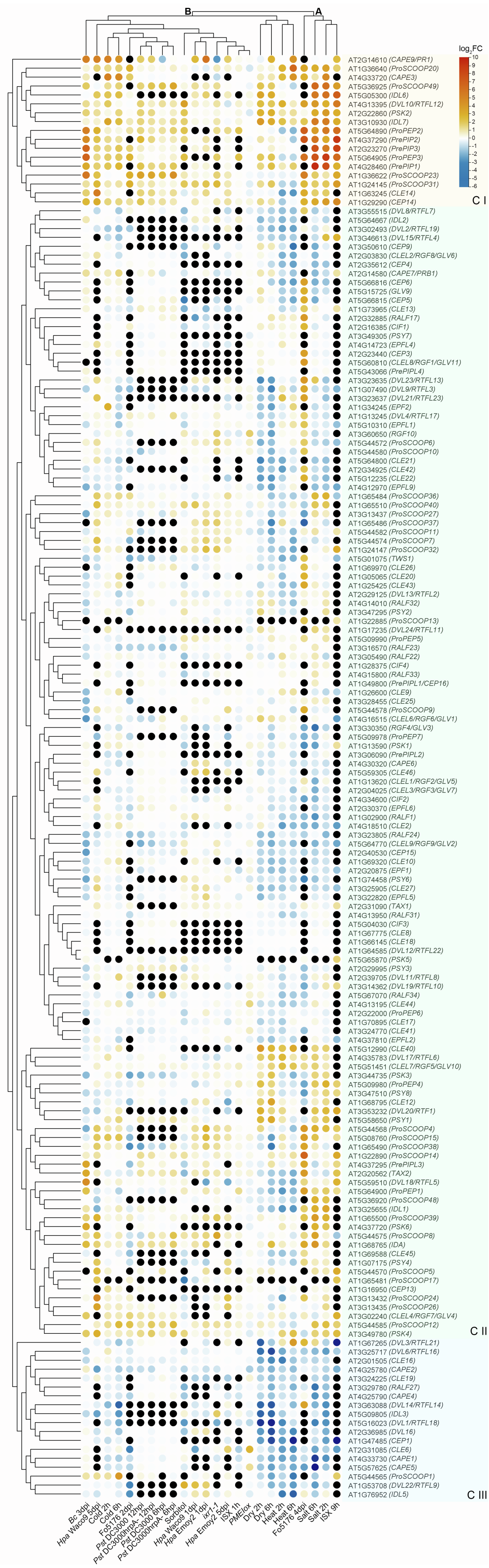

Figure S1. Two-dimensional hierarchical clustering heatmap of peptide precursor-encoding gene expression under abiotic stress, biotic stress, and cell wall modification conditions. Hierarchical clustering was performed on both peptide-encoding genes (rows) and experimental conditions (columns) using the Euclidean distance metric and the complete linkage method (Chen et al., 2020). The resulting dendrograms illustrate relationships among transcripts and conditions. Three major gene clusters [Cluster I (CI), Cluster II (CII) and Cluster III (CIII)] and two condition groups (A and B) are annotated based on their expression patterns. The color scale represents downregulation (blue), no change (white), and upregulation (red). Black squares indicate "Not Available" (NA) values, corresponding to data that are missing for specific conditions within the datasets. Color coding and handling of missing values are described in *Supplemental methods*.
